# Spatio-temporal heterogeneity of metro ridership under major epidemic conditions

**DOI:** 10.1371/journal.pone.0326114

**Published:** 2025-06-17

**Authors:** Baixi Shi, Lijie Yu, Qi Yang, Na Zhang, Nanxi Yang

**Affiliations:** 1 College of Transportation Engineering, Chang’an University, Xi’an, Shaanxi, China; 2 College of Economics and Management, Chang’an University, Xi’an, Shaanxi, China; Tsinghua University, CHINA

## Abstract

The COVID-19 epidemic has significantly altered travelers' behavior, therefore influenced how land use impacts subway ridership. This paper investigates these changes by employing a Geographically and Temporally Weighted Regression (GTWR) model to analyze the spatial and temporal impacts throughout the pandemic. The findings reveal that the outbreak notably reduced metro trip generation across all land use types except residential. Post-pandemic, the influence of workplace, park and green space, and educational land uses in the city center increased. Additionally, workplace land use in rapidly developing areas emerged as a critical factor in boosting metro travel post-epidemic. These insights suggest that commuting, school travel, and outdoor recreation are primary drivers of subway ridership recovery. These results can assist local governments and metro managers in optimizing land use planning and development strategies in the future.

## Introduction

The COVID-19 epidemic, marked by its long duration, high infectivity, and severe symptoms, has significantly altered the travel behavior of urban residents [1]. Travel volumes have declined and fluctuated [2], especially for the public transit [3], due to intermittent and multi-locational outbreaks [4]. There has been a noticeable shift towards private transportation modes. [5]. As a result, in the post-epidemic era, due to the influence of travel habits and psychological fear, travel behavior is hardly to return back to pre-epidemic patterns for a considerable period [6].

The urban rail transit system, as a key public transportation mode, experienced fluctuating ridership during the COVID-19 epidemic [7]. Lockdown policies in many cities limit subway ridership more or less [8]. Even after the resumption of regular metro services, ridership has not fully recovered due to the perceived risk of infection in enclosed spaces [9]. The trips generated by different land use types, or, in other words, for different purposes, have their own recovery efficiency [10,11], considering the impacts of land use on the metro ridership [8,12–14]. The prolonged epidemic and ever-changing containment policies have led to temporary or permanent changes in travel habits [15]. Therefore, it is essential to research the impact of land use on metro ridership throughout the full cycle of the epidemic and understand these changes. Such research can better inform land use planning and development along subway lines in the post-epidemic era.

Many scholars have studied the impact of the COVID-19 epidemic on metro ridership. For instance, several studies have compared metro ridership before and during the epidemic, identifying factors that affect the urban rail transit usage [16–19]. Some others have examined the association between ridership resilience and communities served by stations, considering the impacts of outbreaks and lockdowns [4,20,21].

**Fig 1:**
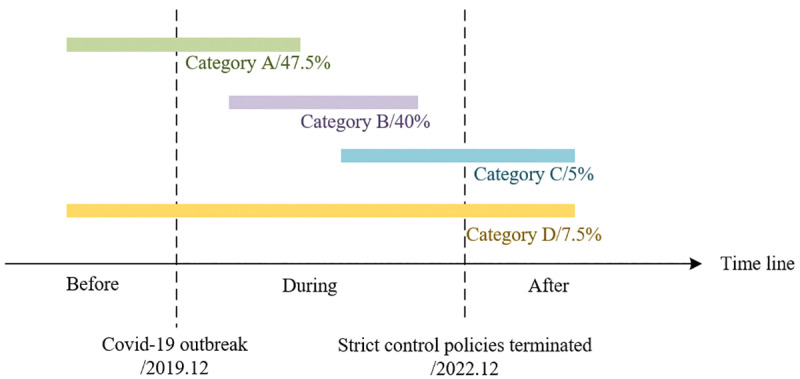
Literature classification by epidemic stages.

Based on the progression of COVID-19 and key time points—such as the outbreak and the lifting of strict control measures—we divide the timeline of the pandemic into three phases: before, during, and after the epidemic. The proportions of references studied in each stage are illustrated in Fig 1. Most studies primarily focus on the periods before and during the epidemic or exclusively analyze research conducted during the epidemic, highlighting early assessments of the virus's impact on ridership and its direct effects. In contrast, only a limited number of studies assess the full cycle of COVID-19, striving for a comprehensive understanding of the virus's long-term effects on metro ridership. Overall, the predominant emphasis is placed on the early and epidemic phases, revealing a notable gap in the literature regarding a holistic perspective on this issue.

On the other hand, the impacts of land use on the metro ridership exhibit spatial-temporal heterogeneity. Studies have found that regions with more active travel are more affected by the epidemic [22]. Stations in the areas with high population density or a high proportion of industrial land use experience fewer changes in ridership due to the outbreaks [16,23,24]. Opposite effects are found regarding the commercial areas [14].

Therefore, this paper focuses on the full cycle of the COVID-19 epidemic and explores its impact on metro ridership based on the Geographically and Temporally Weighted Regression (GTWR) model. The spatial and temporal heterogeneity of these impacts is further analyzed. Data collection, methodology, results, and conclusion are described in the following sections in turn.

## Data collection

By the end of March 2023, Xi'an had operated eight metro lines with the total mileage of 284 km. Given the fact that three of these lines extend to Xianyang, the sub-central city of Shaanxi Province adjacent to Xi’an city, we collect data to analyze the systematic co-evolution of the metro network and the surrounding land use, particularly focusing on the effects of major epidemic condition.

The relevant data applied in this study is as follows.

- **Network Structure Measures of Metro Network**

From the end of 2019 (when the COVID-19 outbreak began) to the early 2023 (when daily metro travels returned to normal level), four metro lines were opened, and three existing lines were extended. To capture the patterns of metro network expansion, we acquired the latitude and longitude of each station in Xi'an from Amap, an online map service, and recorded their opening date accordingly. The network structure measures of the metro network, specifically betweenness centrality(BC), at the end of this year from 2019 to 2023 were calculated to explain the annual variations of station-level ridership. The BC measures the number of shortest paths among all OD pairs on the network that pass through an individual station [25].

- **Station-level ridership**

**Fig 2:**
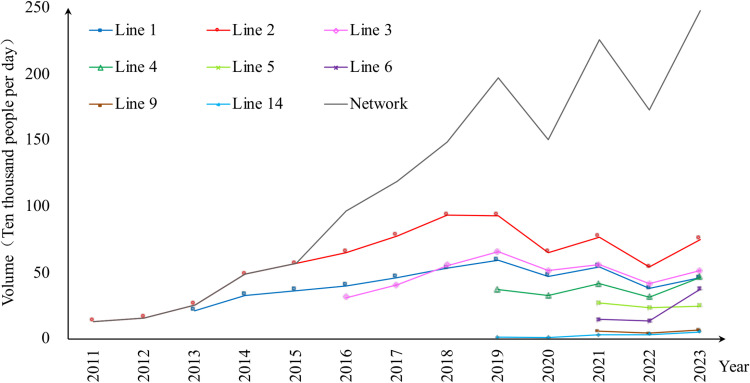
The average annual passenger volume of each metro line and the network in Xi’an from 2011 to 2023 (In 2023, the average volume calculate the first three months).

Daily hourly statistics of station-level ridership were obtained from Xi'an Rail Transit Group Company Limited, which operates the Xi'an metro system. The data includes boardings and alightings at each station, allowing extractions for morning or evening peaks, or aggregation for a full day. We chose the data on weekdays from March of each year from 2019 to 2023 to track the ridership changes before, during and after the COVID-19 outbreak. The annual average passenger volume of each metro line as well as the network overall is shown in Fig 2.

- **Land use**

Points of Interest (POI) within the 1 km catchment area of metro stations were collected from the Amap Open Platform and sorted by year to describe land use development. The overlap between catchment areas was divided according to the Thiessenpolygon method. We classify the POIs into seven types, that are, residential land, commercial land, workplace land, park land, transportation land, educational land, medical land, and cultural land based on residents' travel characteristics.

## Methodology

This section presents the land use attributes used as primary explanatory variables, and introduces the GTWR model to study the spatio-temporal effects of the epidemic on subway ridership.

### Influencing factors

The metro system experienced fluctuating ridership during the COVID-19 epidemic due to lockdowns and ongoing concerns about infection, with varying recovery efficiencies linked to different land use types. These enduring changes in travel habits underscore the necessity of investigating the impacts of land use on metro ridership.

Previous studies have shown that two types of variables have the most significant impact on station-level metro ridership: the built environment within the catchment areas around the stations and demographic characteristics [26]. Among these, the built environment—including attributes related to commercial, residential, educational, medical, parks and other land uses—is regarded as a key factor influencing metro ridership [27–29], even during the pandemic [16,30]. Consequently, land use attributes have been selected as the primary explanatory variables for this study. In this paper, we classify these land use attributes into seven categories: residential land, commercial land, workplace land, park land, transportation land, educational land, medical land, and cultural land, with reference to the official points-of-interest definitions by Amap [31].

Demographic characteristics around metro stations, such as population density [32–34], are also recognized as significant factors affecting metro ridership [26]. Notably, there is a positive correlation between population and residential land use [35]. By analyzing the floor area of residential land, we can estimate the potential population accessing public transportation services, providing a more consistent and tangible representation of the demographic influences on metro ridership.

In addition, network structure variables significantly influence metro ridership. Among these, betweenness centrality is the most important index used to measure the efficiency of metro networks. A station with high betweenness serves as a crucial transit point, facilitating the flow of trips within the metro network [36,37]. Thus, we use betweenness centrality (BC) to represent the importance of stations within the metro network, which can be expressed as [25]:


$$B_{i}=\sum_{s,t=1\left(s\neq t\right)}^{N}\frac{\sigma_{sit}}{\sigma_{st}}$$ (1)

where:

$B_{i}$ is the betweenness centrality of subway station *i*;

$\sigma_{sit}$ is the shortest paths from subway station *s* to station *t* through station *i*;

$\sigma_{st}$ is the shortest paths from subway station *s* to station *t*.

### Model specification

The Ordinary Least Squares (OLS) method is commonly employed to examine factors affecting metro ridership [38–40]. However, it operates under the assumption that the influence of these factors is uniform, which can mask spatial influences and irregularities [27]. Recent research has demonstrated the spatial heterogeneity of various factors and has used the Geographically Weighted Regression (GWR) model for such analyses [41,42]. This spatial influence also persists during the pandemic [43]. Furthermore, given the long-term implications of the pandemic [44,45], this study adopts the Geographically and Temporally Weighted Regression (GTWR) model, which effectively captures both spatial heterogeneity and temporal dynamics [46,47], to investigate how different factors and the surrounding environment affect ridership at various metro stations.

The GWR model can be expressed as [48],


$$y_{i}=\beta_{0,u_{i},v_{i}}+\sum_{k}\beta_{k,u_{i},v_{i}}\cdot x_{ik}+\varepsilon_{i}$$ (2)

where:

$y_{i}$ is the passenger flow of subway station *i*;

$u_{i}$ is the latitude of subway station *i*;

$v_{i}$ is the longitude of subway station *i*;

$x_{ik}$ is the *k* th influencing factor value of subway station *i*;

$\beta_{0,u_{i},v_{i}}$ is the constant term of the regression parameter of subway station *i*;

$\beta_{k,u_{i},v_{i}}$ is the regression parameter of subway station *i* for $x_{ik}$;

$\varepsilon_{i}$ is the error term of subway station *i*.

The GTWR model extends the GWR model by incorporating both spatial and temporal non-stationarity. The GTWR model can be expressed as [49],


$$y_{i}=\beta_{0,u_{i},v_{i},t_{i}}+\sum_{k}\beta_{k,u_{i},v_{i},t_{i}}\cdot x_{ik}+\varepsilon_{i}$$ (3)

where:

$t_{i}$ is the time index of subway station *i*.

The estimates of the regression parameters are,


$$\hat{\beta }={\left(X^{T}WX\right)}^{-1}X^{T}W_{y}$$ (4)

where:

*W* is an *n × n* matrix, in which diagonal elements representing the weight based on the spatial and temporal distance from observation subway station *i*. The off-diagonal elements are zero.

Considering that location and time usually have different scaling effects, the temporal distance is converted into an additional spatial proportional distance. The spatial-temporal distance can be expressed as follows [50]:


$$D_{ij}=S_{ij}⊕T_{ij}$$ (5)

where:

*D*_*ij*_ is the spatial-temporal distance between subway station *i* and *j*;

*S*_*ij*_ is the spatial distance between subway station *i* and *j*;

*T*_*ij*_ is the temporal distance between subway ridership *i* and *j*;

⊕ represents different functions. GTWR model selects “+” to combine spatial distance *S*_*ij*_ and temporal distance *T*_*ij*_ [46]. Hence, spatial-temporal distance between different ridership can be expressed as follows:


$$D_{ij}=S_{ij}+T_{ij}=\sqrt{{\left(u_{i}-u_{j}\right)}^{2}+{\left(v_{i}+v_{j}\right)}^{2}+\tau {\left(t_{i}+t_{j}\right)}^{2}}$$ (6)

where:

*τ* is the spatio-temporal distance ratio.

The diagonal elements of the weight matrix *W* are calculated based on the spatio-temporal distance and a kernel function. There are two commonly used types of spatial kernels: fixed and adaptive kernels [51]. In a fixed kernel function, an optimal spatial kernel is calculated and then applied consistently across the study area. A common weight kernel is a fixed format based on the Gaussian function:


$$\omega_{ij}=exp\left(-{\left(\frac{D_{ij}}{h}\right)}^{2}\right)$$ (7)

where:

$\omega_{ij}$ is the diagonal element of matrix *W*;

*h* is the fixed parameter of space-time bandwidth.

Conversely, the adaptive kernel function identifies a specified number of nearest neighbors to adjust the spatial kernel, maintaining a consistent size for local samples. A commonly used method for adaptive weighting is the bi-square function:


$$\begin{array}{c} \omega_{ij}=\{\;\;\;\;\begin{array}{c} {\left[1-{\left(\frac{D_{ij}}{h_{i}}\right)}^{2}\right]}^{2},\;\;\;if\;D_{ij}<h_{i}\\ \;\;\;\;\;\;\;\;\;\;\;\;\;\;\;\;\;\;0,\;\;\;otherwise \end{array} \end{array}$$ (8)

where:

$h_{i}$ is the different bandwidths, which express the number or proportion of observations to consider in the estimation of regression at station *i*.

The spatio-temporal distance ratio *τ* and the spatio-temporal bandwidth fixed parameter *h* are cross-validated (CV) so that the smallest *τ* and *h* are determined as [52],


$$C=\sum_{i=1}^{n}{\left(y_{i}-{\hat{y}}_{\neq i}\right)}^{2}$$ (9)

where:

*C* is the value obtained by cross validation;

*n* is the total number of subway stations;

${\hat{y}}_{\neq i}$ is the estimates of the GTWR model based on the training data excluding station *i*, which is a function of *τ* and *h*.

In practice, plotting the CV against the parameter *h* can help determine a suitable value for this parameter. Alternatively, an appropriate value can be automatically derived using an optimization technique that minimizes Equation (9) based on goodness-of-fit statistics or the corrected Akaike Information Criterion (AICc) [53].

## Results and analysis

### Model comparison

#### Comparison of different models.

After conducting the collinearity test, five types of land use attributes are remained in our models: workplace land, educational land, residential land, medical land, and park land, following the stepwise regression method. For each year, encompassing both weekdays and weekends, we select the same independent variables to construct the OLS, GWR, and GTWR models. We employ the GTWR ADD-IN developed by Huang et al. [46] to fit these three models. In the analysis of the GWR and GTWR models, we compare both fixed and adaptive kernels, along with AICc and CV bandwidths. The spatio-temporal distance ratio (τ) is determined using an automatic optimization method. The fitting results are presented in Tables 1 and 2.

**Table 1:** Fitting results of OLS, GWR, and GTWR for weekday.

Model	Kernel	Bandwidth	RSS	AICc	Adjusted R^2^
**OLS**	–	–	6.67E + 10	1.41E + 04	0.366
**GWR**	Fixed	AICc	5.36E + 10	1.40E + 04	0.487
	Fixed	CV	5.36E + 10	1.40E + 04	0.487
	Adaptive	AICc	4.54E + 10	1.40E + 04	0.566
	Adaptive	CV	4.54E + 10	1.40E + 04	0.566
**GTWR**	Fixed	AICc	3.45E + 10	1.38E + 04	0.670
	Fixed	CV	3.45E + 10	1.38E + 04	0.670
	Adaptive	AICc	3.20E + 10	1.38E + 04	0.694
	Adaptive	CV	3.18E + 10	1.38E + 04	0.695

**Table 2:** Fitting results of OLS, GWR, and GTWR for weekend.

Model	Kernel	Bandwidth	RSS	AICc	Adjusted R^2^
**OLS**	–	–	9.62E + 10	1.43E + 04	0.288
**GWR**	Fixed	AICc	7.77E + 10	1.43E + 04	0.421
	Fixed	CV	7.77E + 10	1.43E + 04	0.421
	Adaptive	AICc	6.73E + 10	1.42E + 04	0.498
	Adaptive	CV	6.73E + 10	1.42E + 04	0.498
**GTWR**	Fixed	AICc	5.75E + 10	1.42E + 04	0.572
	Fixed	CV	5.75E + 10	1.42E + 04	0.572
	Adaptive	AICc	5.38E + 10	1.42E + 04	0.599
	Adaptive	CV	6.63E + 10	1.42E + 04	0.506

Table 1 presents the fitting results of the OLS, GWR, and GTWR models for weekdays. The OLS model shows an adjusted R^2^ value of 0.366. For the GWR model, the best results correspond to the adaptive kernel, which achieves an adjusted R^2^ value of 0.566. The GTWR model outperforms the GWR, achieving the highest R^2^ of 0.695 when using adaptive kernels with CV bandwidth.

Table 2 provides similar fitting results for the OLS, GWR, and GTWR models for weekends. The OLS model shows an adjusted R^2^ of 0.288. For the GWR model, the adaptive kernels perform better, with an adjusted R^2^ of 0.498. In the GTWR results, the best performance is achieved with the adaptive kernel AICc, yielding an adjusted R^2^ of 0.599.

Overall, both tables illustrate that the GTWR model generally provides superior fitting statistics compared to OLS and GWR, with adaptive kernels yielding enhanced performance across both weekdays and weekends. Consequently, we select the adaptive kernel with AICc bandwidth for further analysis.

#### Sensitivity analysis of parameters in GTWR model.

**Fig 3:**
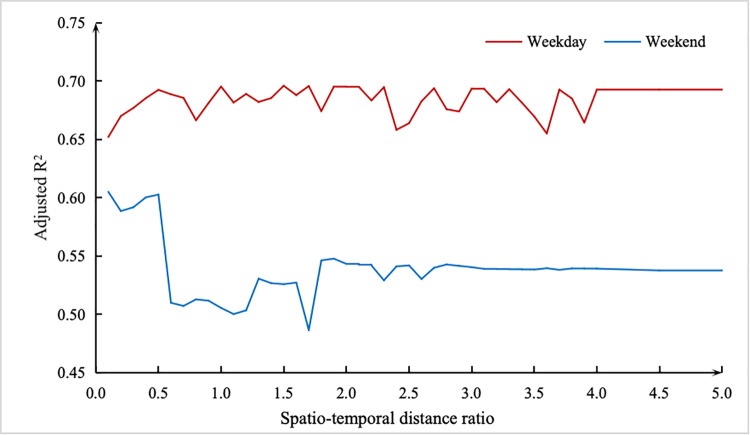
The mean fitting coefficient of the independent variablesTemporal effects.

According to Huang’s study [46], only the spatio-temporal distance ratio *τ* plays an important role in constructing weights. Hence, the parameter *τ* needs to be determined. Fig 3 presents the parameters *τ* and adjusted R^2^ in the situation of adaptive kernels with CV bandwidth. We can see that overall, the R^2^ values fluctuate at different *τ*. However, when the *τ* exceeds 4.1, the R^2^ values for both weekdays and weekends stabilize. Furthermore, the R^2^ value on weekdays is significantly higher than that on weekends, indicating that the model fits better during weekdays compared to weekends.

Compared to the GTWR results presented in Tables 1 and 2, the model established using the automatic optimization method to determine the *τ* value achieves a relatively high R^2^ value. Consequently, subsequent analyses will continue to build upon this result.

**Fig 4:**
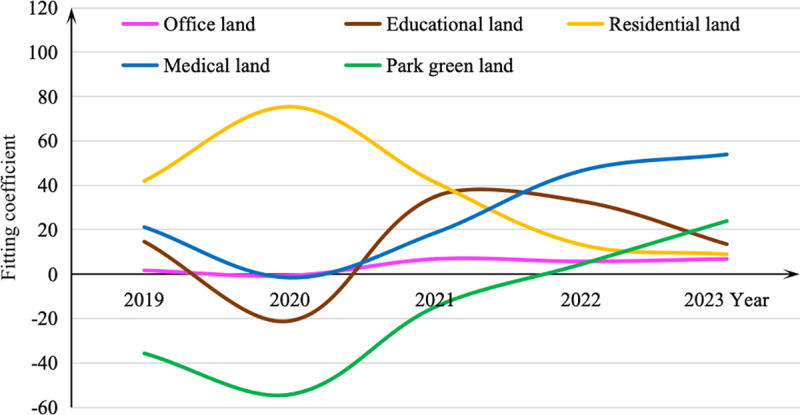
The mean fitting coefficient of the independent variables in weekday.

**Fig 5:**
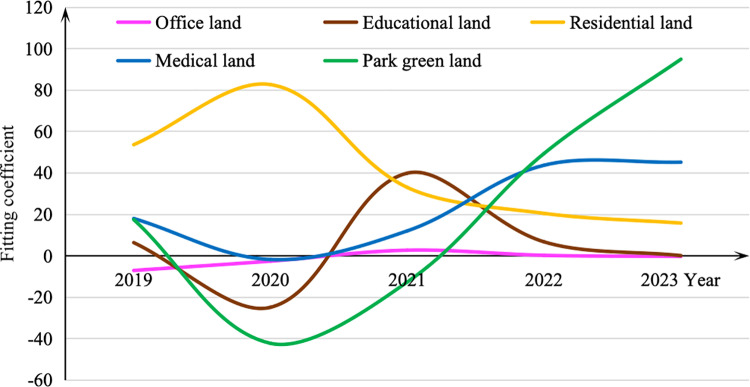
The mean fitting coefficient of the independent variables in weekend.

The mean values of the coefficients for the independent variables in each year are summarized in Table 3, and its trend of change is shown in Figs 4 to 5.

**Table 3:** The mean fitting coefficient of the independent variables for each year.

Time	Independent variable	2019 year	2020 year	2021 year	2022 year	2023 year
**Weekday**	Workplace	1.700·	−0.64	6.87	5.75	6.840·
	Educational	14.650·	−21.05	35.040**	32.840***	13.490·
	Residential	42.000·	75.390**	41.300***	13.290***	8.930*
	Medical	21.210*	−1.55	18.67	46.55	53.940·
	Parks	−35.68	−54.21	−14.69	4.46	23.97
	BC	3.12E + 04**	−1.57E + 03	1.18E + 04	2.21E + 04**	3.54E + 04***
**Weekend**	Workplace	−7.03	−2.55	2.73	0.26	−0.27
	Educational	6.380·	−24.82	39.920**	6.750**	0.12
	Residential	53.670·	82.650*	33.020**	20.500***	15.820*
	Medical	18.030·	−1.75	12.07	43.74	45.2
	Parks	17.470·	−42.21	−12.6	49.45	94.910*
	BC	3.55E + 04**	7.47E + 02	1.60E + 04	1.69E + 04·	4.41E + 04***

*** p-value<0.001, ** p-value<0.01, * p-value<0.05,*·* p-value <0.1

As it shows, the independent variables have significant influences on the ridership in some of the selected years (none of them are statistically significant in all years) except for park greenspace land use on weekdays and workplace-related factors on weekends. This is quite reasonable as commuters barely have time for entertaining or sporting in the parks during the weekdays and workplaces are not attractive for them as well during the weekends.

Considering the temporal trends during the full cycle of the COVID-19 epidemic, we can find that the estimates of other non-residential independent variables reach the minimum in the year of 2020, which is the start of the COVID-19 outbreak. While in the period from 2021 to 2022 when the confirmed cases were reported discontinuously, the influence of residential and educational land use stand out. An interesting phenomenon is that the effects of medical and park green land use are much improved after the epidemic compared to the year before, maybe because people pay more attention to exercising or playing in the open space for maintaining good health. All these indicate that the epidemic has really brought a huge impact on public transit travels.

The effects of the residential land use have a different temporal trends that its coefficient reaches the peak in 2020. It implies that the regular travel is restricted due to the strict control policy during the peak period of COVID-19 outbreak and the home-based trips, e.g., to hospitals or to supermarkets, are dominating in order to guarantee the daily needs. While at the end of the epidemic, the degree of such an impact are gradually decreased, even lower than the time before the epidemic. This may be because with the expansion of the metro network, residents have more choice to go to the places they want using the metro, and relatively, the impact of the residential land use declines.

Educational land use shows a notable drop in influence in 2020 aligns with the widespread closures and online learning adaptations due to the pandemic, demonstrating how institutional closures significantly impacted metro ridership during that period. A marked increase in influence from 2021 to 2022, which suggests that as the public adapted to new routines post-lockdown, the demand for educational activities surged. Educational facilities became more significant in shaping metro ridership patterns as schools and universities reopened and resumed normal operations.

Workplace is a typical land use type for commute travels, as it has a positive effect on weekdays and a negative impact on weekends in the normal years, e.g., 2019 or 2023. But the workplace areas restrain the ridership during the outbreak, like many other non-residential land use types. Moreover, the impact after 2020 is higher than that in 2019. Since the longtime strict control policy in 2020, business activities such as private companies and individual households have been experiencing a hard time. So that during 2021–2023, the income reduction push more residents to go out for working.

Medical land use has a significant decline in ridership related to medical trips in the early pandemic year of 2020, likely because of fears surrounding the virus and restrictions on non-essential medical services. However, it exhibits improvements in its impact after the COVID-19, as suggested by the higher coefficients reported in 2022 and 2023 compared to 2019. This increase is likely due to a heightened public awareness of health and wellness. As people resumed more regular healthcare visits, the influence of medical land use became an essential factor in public transit patterns.

Park green land use shows a contrasting trend throughout the pandemic. Initially, it experienced a considerable decline in influence during 2020, reflecting restrictions on social gatherings and outdoor activities. However, post-pandemic data indicates a significant resurgence in the use of parks and green spaces, leading to a positive impact on ridership by 2023. This change correlates with the public's increased desire for outdoor recreation and exercise as a means of coping with stress and maintaining physical health, demonstrating a shift toward valuing open spaces in urban planning and public transit considerations. The higher coefficients in subsequent years suggest that parks became vital destinations for residents seeking leisure and well-being after the pandemic, thus enhancing their influence on ridership.

### Spatial effects

From the perspective of space, we analyzed the distribution of the impact of various types of land use on metro ridership, and compared the difference of the spatial distribution of the impact of each land use on passenger flow in 2023 and 2019 from the perspective of time.

#### Current situation analysis.

**Fig 6:**
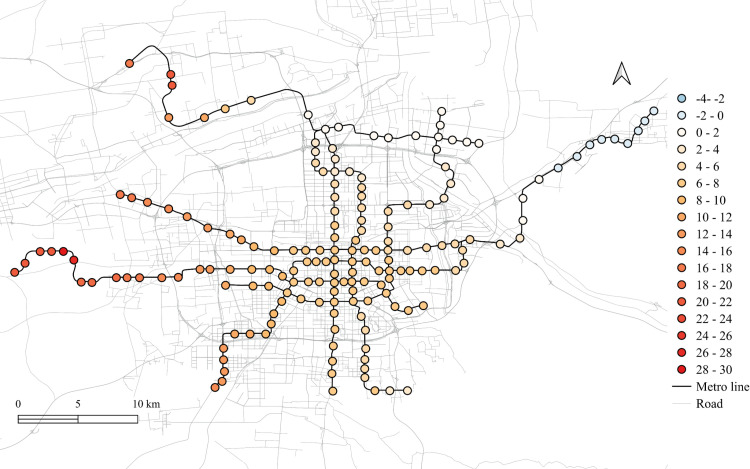
The spatial fitting results for workplace areas in weekday of 2023.

**Fig 7:**
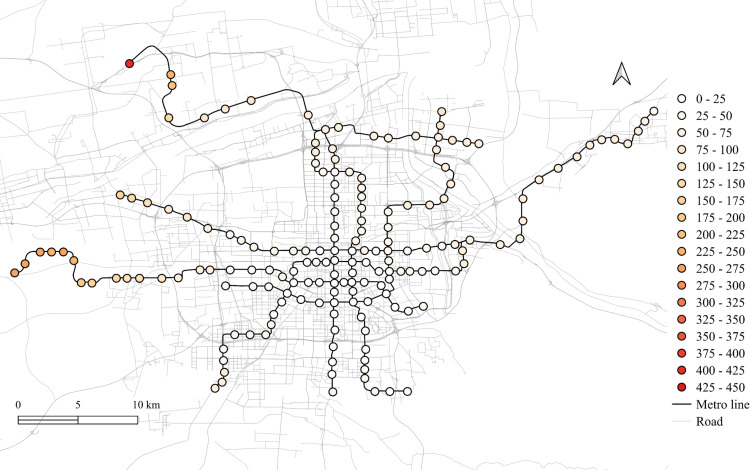
The spatial fitting results for medical land in weekday of 2023.

**Fig 8:**
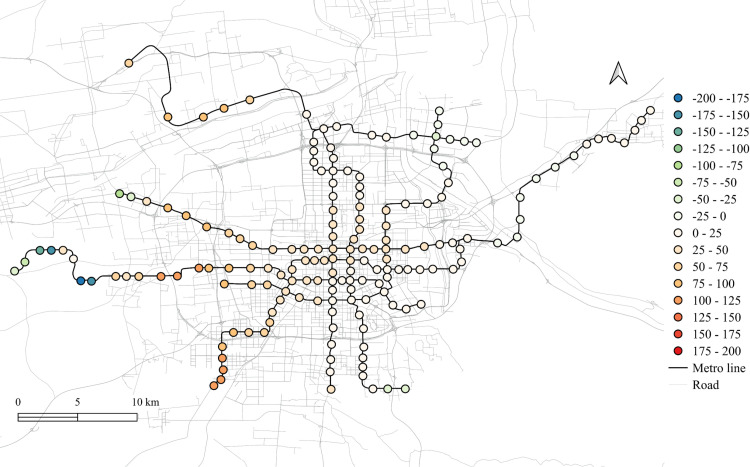
The spatial fitting results for park and green space in weekday of 2023.

**Fig 9:**
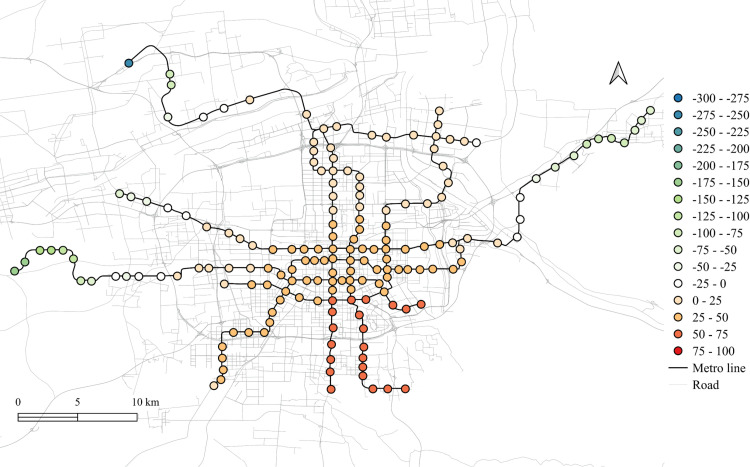
The spatial fitting results for education land in weekday of 2023.

**Fig 10:**
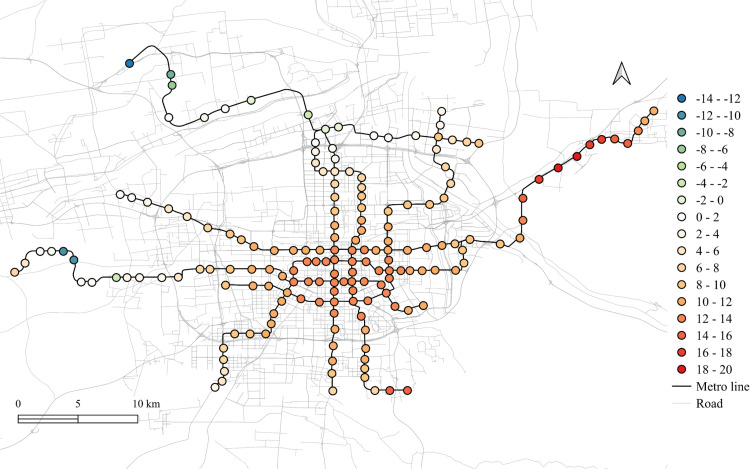
The spatial fitting results for residential land in weekday of 2023.

The spatial distribution for the regression coefficients for different lands on weekdays in 2023 is shown in Figs 6–10 in which the cold colors represent negative coefficients, while the warm colors express positive ones.

The impacts of workplace areas on ridership are mainly positive and deepen from the west to the east, as shown in Fig 6. Great impact of workplace areas also concentrate in the western region, which is in accordance with the development orientation of the Xi’an city in recent few years. Stations near the city center is less affected by the workplace land use, probably because of the highly mixed land use nearby. While the east is dominated by the residential and tourism spots, such as the Terracotta Warriors and the Huaqing Pool, thus, the westward development became inevitable, to the workplace land use contributes less to the subway ridership in this area.

The residential land use in the city center has a higher impact on station passenger flow and attenuated to the marginal area, as shown in Fig 10. But note that the stations in the eastern periphery also remain much effects on the ridership, for which the corresponding metro line reaches an isolated sub-city of Xi’an, so commute travels via the subways are dominated by the residential land use nearby. In addition, it cannot be ignored that the spatial distributions of the employment and residential locations are not balanced, comparing Figs 6 and 10, which makes the west-east corridor be the main direction of commute travels. Future plans should pay attention to the transport supply and demand equilibrium on this corridor.

Among other types of land use, the medical land use also promotes the growth of subway ridership overall, but not as outstanding as the workplace, particularly for the stations within the Third Ring Road (the well developed area in relative). The impact degree increases from the middle to the periphery, especially in the western region, as shown in Fig 7. This implies that new zones pay more attention to the supporting of medical land use development. The park and green space also has a relatively high impact on the mid-western region, while a sharp decline can be found in the western exurban, see Fig 8. Some famous tourist attractions, such as the Bell Tower, the Wild Goose Pagoda, and the Huaqing Pool near the city center and the Terra Cotta Warriors in the east, do not show a significant impact on metro ridership, even though the passenger flows of their nearest stations are at a relative higher level. This may suggests that the park and green space can promote ridership when the land-use development around the station is not mature, while in the developed area, this kind of land use is not the key to improve ridership. The greatest influence of the education land use are found in the city center as well as the southern area, as shown in Fig 9, which is expected as the cultural and educational resources are gathered there.

#### Temporal variation analysis.

**Fig 11:**
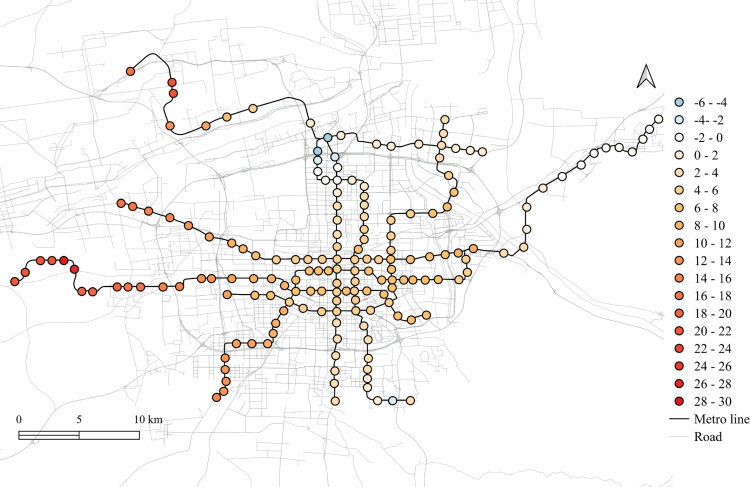
The differentiation of spatial fitting results for workplace areas in workday between 2023 and 2019.

**Fig 12:**
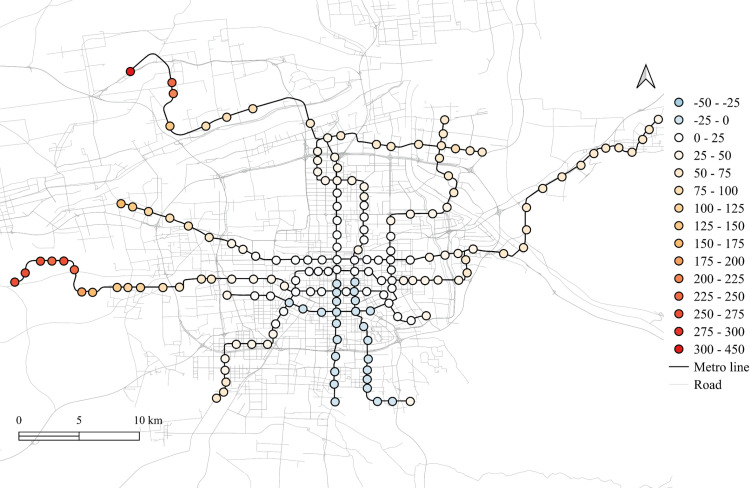
The differentiation of spatial fitting results for medical land in workday between 2023 and 2019.

**Fig 13:**
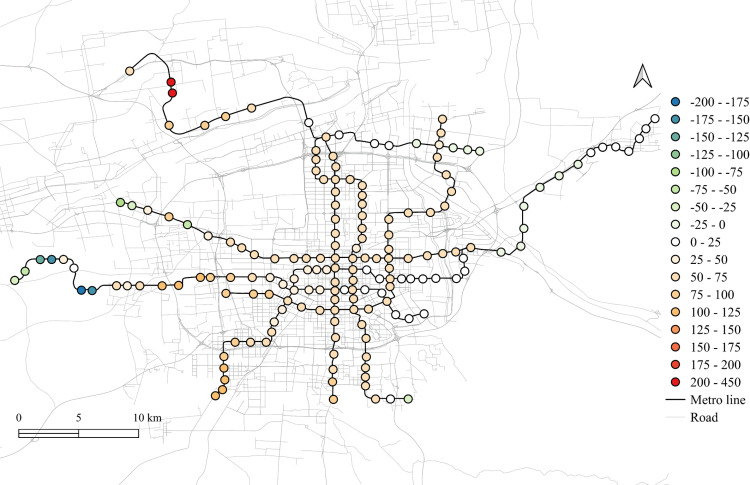
The differentiation of spatial fitting results for park and green space in workday between 2023 and 2019.

**Fig 14:**
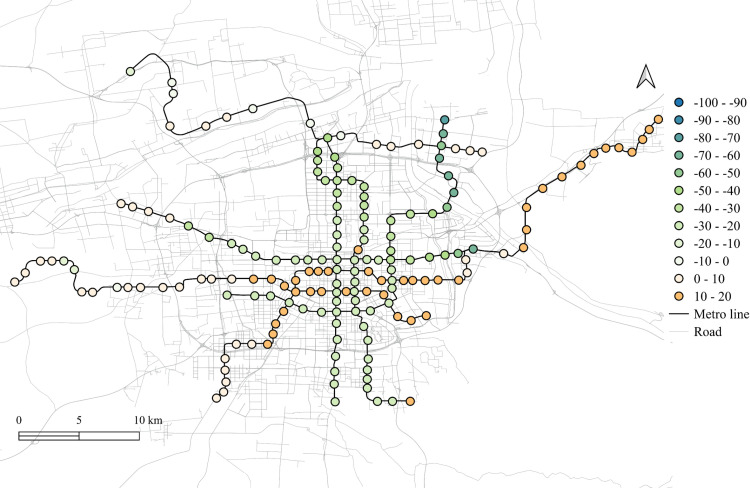
The differentiation of spatial fitting results for education land in workday between 2023 and 2019.

**Fig 15:**
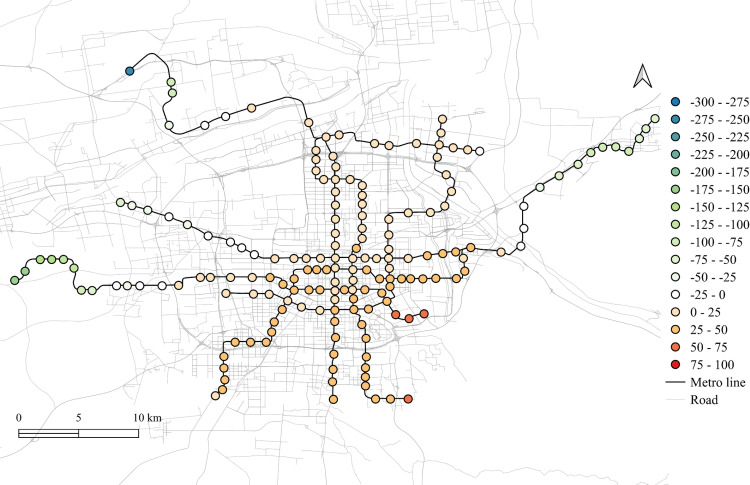
The differentiation of spatial fitting results for residential land in workday between 2023 and 2019.

The differences of the regression coefficients on weekdays between 2023 and 2019 are shown in Figs 11–15. Samely, the cold colors represent the negative impacts, while the warm colors expresses the positive ones.

It clearly shows that people's travel behaviors are truly affected by the epidemic, comparing the before and post epidemic scenarios. For the workplace, park and green space, as well as the educational land use, the degree of its impact on metro ridership are intensified to the city center. This indicates that work and school travels attracted by office and education land use may become the main attributes for ridership recovery.

The increase in the impact of the park and green space in many scopes of the city shows that residents have a positive attitude towards open spaces in the post-epidemic scenario, compared to the before-epidemic scenario. And at the same time, the no exhibit spatial aggregation reflects the randomness of leisure flow.

However, the residential and medical land use are less attractive to ridership between 2023 and 2019. Except for the three new lines, the influence of residential land use decreases in the post-epidemic scenario. Especially in the northeast area around the logistics distribution center, whose population is small, the phenomenon of decline is more obvious. While the effect for medical land use reduce not much and the main reduction area is in the southern region.

## Conclusion

This paper investigates the impact of the COVID-19 epidemic on metro ridership by considering the full cycle of the pandemic, from its onset to the post-pandemic recovery phase. Compared to the OLS and GWR models, the GTWR model shows its superiority in fittings. Its R^2^ values are much higher than the other two for both the weekday and weekend scenarios. This demonstrates the intrinsic spatial and temporal correlations of metro ridership at the station level.

The temporal trends identified from the GTWR models indicate that the epidemic has brought a huge impact on travels via subways. To be more specific, the coefficients of all selected independent variables except the residential land use reach the minimum value in 2020, the start of the COVID-19 outbreak, which highlights its negative externality. But for the residential land use, it shows its importance when explaining the metro ridership even during the outbreak year, and interestingly, its coefficient goes to the peak in 2020, which implies that the home-based rigid demand dominates the metro passenger flows, compared to other land use types.

During the unstable period, from 2021 to 2022, when intermittent and small-scale outbreaks could be detected, the impacts of educational land use recover due to the cancellation of the national strict control policy. The effects of the workplace get back as well, even surpass the pre-pandemic level, which supports the findings from Ha’ study [14]. While in the post-pandemic period, positive effects of medical and park green land use on station-level ridership can be observed probably because people pay more attention to healthy lifestyle after the epidemic.

Spatial heterogeneity is also detected for the impacts of different land use attributes on the metro ridership. The influences of workplace, park and green space, and educational land use in Xi'an city center increase in the post-pandemic scenario, which tells that commute, student, and outdoor recreational travels might be the main attributes for subway ridership recovery. It is worth noting that the impact of workplace land use on the metro usage in the western region of Xi'an is higher than the city center. As a rapidly developing region in the recent few years, it is understandable as the recovery of economic activities is a crucial development objective after the epidemic [30].

Such results help to explain the relationship between land uses and metro usage, and some policy implications are revealed. The decline in metro ridership when the epidemic outbreak is inevitable [54,55], but financial losses can be reduced by cutting the departure frequency. When the government partially repeals the controls, the frequency can be gradually increased and priority should give to the city center or new built-up areas. In addition, as the impact of the medical and green & park land uses have increased dramatically in the post-pandemic scenario, we need to balance the spatial distribution of these two types of land use in the urban area, so as to promote the utilization efficiency of the metro system [56].

This study is based on comprehensive metro ridership data from Xi'an, accurately reflecting the local situation. However, it is important to note that different cities may exhibit distinct ridership patterns. Therefore, the specific findings drawn from this research are primarily relevant to Xi'an and may not be directly applicable to other urban contexts. Nonetheless, our overall modeling approach, particularly the use of the GTWR model to analyze the impact of the built environment on the spatial and temporal changes in metro ridership, can be adapted for application in other cities, provided they have similar detailed data support.

Our paper is primarily a practical study rather than a methodological one. It investigates how various factors influence metro ridership throughout the full cycle of epidemic. To accomplish this, we selected an appropriate method for our analysis. For future studies aimed at improving the accuracy of ridership predictions, there is significant potential to develop new models that could provide a more precise examination of these issues.

## Supporting information

S1 Data xlsx. (XLSX)
